# Differential impacts of interstitial lung disease and airway disease on rheumatoid arthritis disease activity and infection

**DOI:** 10.1038/s41598-025-23080-1

**Published:** 2025-11-13

**Authors:** Misaki Yoshida, Midori Ueno, Takeshi Zoshima, Dai Inoue, Ichiro Mizushima, Satoshi Watanabe, Seiji Yano, Hideki Nomura, Mitsuhiro Kawano

**Affiliations:** 1 Department of Nephrology and Rheumatology, Graduate School of Medical Sciences, Kanazawa University, Kanazawa, Japan; 2 Department of Radiology, Kanazawa University Hospital, Kanazawa, Japan; 3 Department of Respiratory Medicine, Kanazawa University Hospital, Kanazawa, Japan; 4 Department of General Medicine, Kanazawa University Hospital, Kanazawa, Japan; 5 Department of Hematology and Immunology, Kanazawa Medical University, 1-1, Daigaku,, Uchinada, Kahoku-gun, Ishikawa 920-0293 Japan

**Keywords:** Rheumatoid arthritis, Interstitial lung disease, Bronchitis, Rheumatology, Risk factors

## Abstract

We aimed to distinguish the individual effects of interstitial lung disease (ILD) and airway disease (AD) on rheumatoid arthritis (RA) disease activity and infection. RA patients who underwent chest CT at Kanazawa University Hospital (2011–2021) were investigated. The primary outcome was infection-related hospitalization, and the secondary was RA disease activity (DAS28-CRP, SDAI) at final observation. Of 569 patients, 125 (22.0%) had ILD (ILD +), and 171 (30.1%) had AD (AD +). At first CT: RA disease activity was similar; ILD + received less methotrexate than ILD–; all DMARDs were similar in AD + and AD–. At final observation (mean: 73.9 months): RA disease activity was higher in ILD + and AD + . Analysis of covariance revealed a significant association between final RA disease activity and ILD, but not AD. Kaplan–Meier analysis showed lower infection-free rates in ILD + and AD + . Cox regression analysis with time-dependent covariates showed an association between infection and ILD after 50 months and AD (HR 3.702 and 1.744). RA disease activity was independently associated with ILD, likely due to reduced methotrexate use. ILD after 50 months and AD both increased infection risk independently. Distinguishing ILD from AD is important for selecting safe and effective treatments.

**Supplementary Information:**

The online version contains supplementary material available at 10.1038/s41598-025-23080-1.

## Introduction

Rheumatoid arthritis (RA) frequently presents with extra-articular manifestations, including pulmonary involvement, which is detected in 60–80% of cases accounting for 10–20% of all-cause mortality attributed to pulmonary factors^1^. Of these, interstitial lung disease (ILD) affecting the parenchyma, and lower airway disease (AD) involving the lower airways, are significant.

Highly prevalent forms of ILD include unusual interstitial pneumonia (UIP), nonspecific interstitial pneumonia (NSIP), and organizing pneumonia (OP). Rarer lesions include lymphocytic interstitial pneumonia, acute interstitial pneumonia, and diffuse alveolar damage^2,3^. Occurrence of ILD in RA patients varies from 5 to 67%^4–7^. This wide range may be attributed to the often non-symptomatic nature of RA associated ILD^8^. The predominant type of AD is bronchiectasis, followed by follicular bronchiolitis and constrictive bronchiolitis^3^. AD is detected in 20–60% of RA cases^9–13^.

Lung-related complications are a major cause of mortality in RA^14–16^. Age-adjusted mortality rates in 2018 matched those in 2005, signifying a lack of improvement for patients with RA and ILD, despite positive trends in RA patients overall. Consequently, management of lung lesions in RA remains in need^2,17^.

A key mechanism through which lung complications in RA may impact patient outcomes is heightened risk of severe infections. RA-associated ILD may increase infection risk due to pulmonary involvement, compromised immune defense in lungs, and use of immunosuppressive therapy for pulmonary conditions^18,19^. Similarly, RA-associated AD may increase infection risk and is associated with poorer prognosis^2,20–22^. When patients with high risk of infection due to lung complications require immunosuppressive anti-rheumatic drugs for disease control, clinicians may hesitate to make treatment decisions.

When ILD and AD coexist in RA patients, the cumulative risk of infection and mortality becomes a significant concern. However, reports on the clinical characteristics and prognoses of RA-associated ILD and AD have not examined their independent effects on the clinical course of RA. The presence or absence of an interaction between ILD and AD remains unclear.

We conducted a retrospective hospital cohort study to investigate the independent effects of ILD, AD, and any interactions between them on RA disease activity and development of infections.

## Methods

### Patients

We retrospectively evaluated RA patients treated at Kanazawa University Hospital Rheumatology Department between April 2011 and March 2021. RA was diagnosed based on the 1987 American College of Rheumatology (ACR)^23^ or the 2010 ACR / The European Alliance of Associations for Rheumatology (EULAR) criteria^24^. Patients with incomplete evaluation of RA-associated lung lesions on chest CT scans due to factors such as prior or current respiratory infections were excluded. The study adhered to the tennets of the Declaration of Helsinki and was reviewed and approved by our university Ethics Committee (approval number 2021–319). Due to the retrospective nature of the study, informed consent was waived by our university Ethics Committee (approval number 2021-319).

### Clinical assessment

We collected demographic, clinical, and laboratory data from medical records. Baseline data was that obtained at time of initial chest CT scan and follow-up period was until the final hospital visit observation.

### Interpretation of chest CT scan images

To ascertain the presence or absence of ILD and AD, chest CT images were visually evaluated by two experienced radiologists (M.U. and D.I.) blinded to all clinical data. The major radiographic patterns have been identified in patients with RA-ILD as follows: UIP pattern; NSIP pattern; OP pattern^3^. We defined UIP pattern as bilateral subpleural reticulation with or without honeycombing, NSIP pattern as predominant ground-glass opacities, and OP pattern as patchy areas of consolidation, based on a previous report^3^. AD was defined as centrilobular nodules, tree-in-bud patterns, bronchial wall thickening, bronchiectasis, bronchiolectasis, mucoid impaction, and mosaic attenuation, as described^3,25,26^. CT findings of bronchiolitis were defined as centrilobular nodule, tree-in-bud patterns, bronchiolitis, and mosaic attenuation^26^. In each patient, predominant CT patterns were defined, and the most likely CT diagnosis was determined^3^. ILD was categorized as either UIP or non-UIP, which included NSIP and OP. AD was categorized as either bronchiolitis or other forms.

Based on chest CT findings, patients were classified into groups with ILD (ILD +) and without ILD (ILD¬), and with AD (AD +) and without AD (AD–). Combinations included four subgroups: ILD–AD–, ILD–AD + , ILD + AD–, and ILD + AD + .

### Primary and secondary outcome measures

The primary outcome was time to first infectious event requiring hospitalization, defined as microbiologically proven or clinically diagnosed infection anywhere on the body. The secondary outcome was RA disease activity assessed by Disease Activity Score 28 based on C-reactive protein (DAS28-CRP) or Simplified Disease Activity Index (SDAI) at final observation.

### Statistical analyses

Continuous variables were presented as mean ± SD with 95% confidence intervals (C.I.). We used Mann–Whitney U test to compare means between two groups and Kruskal–Wallis test followed by pairwise Mann–Whitney U with Bonferroni’s correction for comparisons among four groups. Categorical variables were presented as counts, percentages and 95% C.I. Proportions were compared using Pearson’s chi-square or Fisher’s exact test. Post hoc pairwise z-tests with Bonferroni’s correction were used for comparisons among four groups. Wilcoxon signed-rank sum test was used to compare continuous variables at baseline with those at final observation.

Kaplan–Meier method was used to estimate cumulative infectious event-free rates at different time points. The stratified log-rank test was used to assess group differences, and Cox’s proportional hazards model was used to compare time to event. If an interaction between two variables showed no significant effect, re-analysis was performed without that interaction. When deemed appropriate, we applied Cox proportional hazards analysis with time-dependent covariates. Effect sizes were presented as hazard ratios (HR) and 95% C.I.

Two-way analysis of covariance (ANCOVA) was used to compare continuous variables between 2 × 2 groups, with covariates identified as significant factors affecting the dependent variable. Non-significant interactions between independent variables were removed from analysis. Estimated marginal means of disease activity indices in ILD + and ILD– groups, and AD + and AD– groups, along with their differences, were described with 95% C.I. Dependent variables were log-transformed when necessary. In such cases, estimated marginal (arithmetic) means of the log-transformed variables were back-transformed to geometric means in their original scale, and their differences were expressed as geometric mean ratios.

All statistical analyses were performed using SPSS for Windows (Version 27.0, IBM Corp. Armonk, NY, USA) with p < 0.05 deemed significant. The details are shown in Supplemental Methods.

## Results

### Enrolled patients

**Fig. 1:**
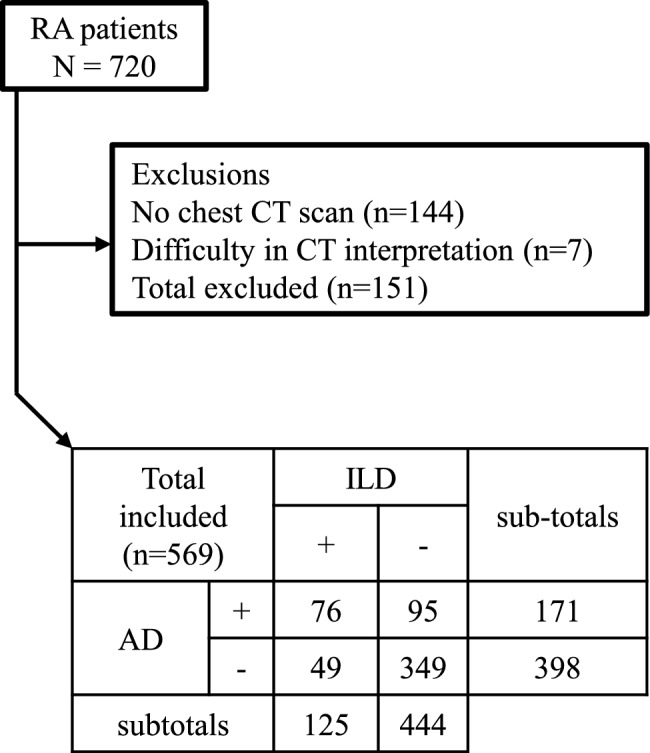
Schematic representation of the study cohort. Enrolled patients with RA classified into groups with and without interstitial lung disease (ILD), and with and without airway disease (AD) and their combinations.

Of 720 patients identified with RA, 144 were excluded due to absence of chest CT images and seven due to difficulty interpreting CT findings leaving 569 for analysis (Fig. 1). Chest CT scans were performed for reasons including screening for respiratory comorbidities, tracking RA-associated lung lesions, and assessing other conditions with or without respiratory symptoms. Among the 569 patients, 125 (22.0%) had ILD, 171 (30.1%) AD, and 76 (13.4%) both (Fig. 1). UIP was observed in 35 (28.0%) of ILD + group and bronchiolitis in 79 (46.2%) of AD + group. Interestingly, 60.9% of ILD + group had AD, whereas 44.5% of AD + group had ILD. The mean ± SD observation period for all patients was 73.9 ± 57.3 months.

### Patient characteristics at baseline

**Fig. 2:**
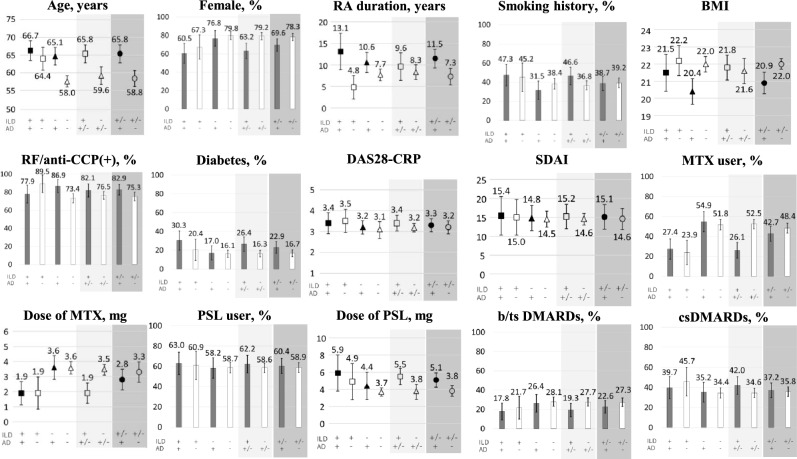
Patient characteristics at baseline. Characteristics of patient groups at baseline including proportions, means, and 95% confidence intervals. Abbreviations: RA (rheumatoid arthritis), BMI (body mass index), PSL (prednisolone), MTX (methotrexate), bDMARDs/tsDMARDs (biologic disease modified anti-rheumatic-drugs / targeted synthetic DMARDs), csDMARDs (conventional synthetic DMARDs), RF/anti-CCP( +) (rheumatoid factor and/or anti-CCP antibodies), DAS28-CRP (disease activity score-28 using C-reactive protein), SDAI (simplified disease activity index).

Baseline characteristics of patients are shown in Fig. 2 and Table 1. The mean ± SD age was 60.9 ± 14.1 years with none aged under 20 at time of first chest CT scan. Females comprised 75.7%. The mean ± SD RA disease duration was 8.6 ± 13.2 years. Notably, 77.8% of patients tested positive for rheumatoid factor (RF) and/or anti-CCP antibodies. DAS28-CRP and SDAI scores were 3.2 ± 1.4 and 14.7 ± 12.3, respectively. Use of methotrexate (MTX), glucocorticoids, and biologic disease modified anti-rheumatic-drugs (bDMARDs)/targeted synthetic DMARDs (tsDMARDs) was observed in 46.9%, 59.5%, and 25.9% of patients, respectively.

**Table 1:**
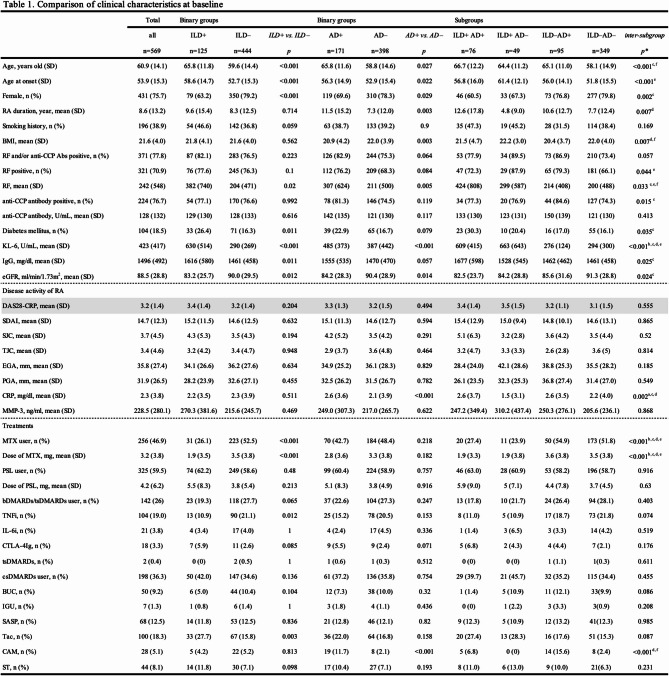
Comparison of clinical characteristics at baseline.

ILD + group was older than ILD– group (65.8 ± 11.8 vs. 59.6 ± 14.4 years, *p* < 0.001), had a lower proportion of females (63.2 vs. 79.2%, *p* < 0.001), higher incidence of AD (60.8 vs. 21.5%, *p* < 0.001), and higher prevalence of diabetes (26.4 vs. 16.3%, *p* = 0.013). They had higher serum levels KL-6 (630 ± 514 vs. 290 ± 269 U/ml, *p* < 0.001) and IgG (1616 ± 580 vs. 1461 ± 458 mg/ml, *p* = 0.011), and a lower estimated glomerular filtration rate (eGFR) (83.2 ± 25.7 vs. 90.0 ± 29.5 ml/min/1.73m^2^, *p* = 0.012). No significant difference was found in DAS28-CRP or SDAI scores between ILD + and ILD– groups. Use of MTX was less common in ILD + group (26.1 vs. 52.5%, *p* < 0.001; 1.9 ± 3.5 vs. 3.5 ± 3.8 mg, *p* < 0.001), while use of glucocorticoids and conventional synthetic DMARDs (csDMARDs), and bDMARDs/tsDMARDs was similar between groups.

AD + group was older than AD– (65.8 ± 11.6 vs. 58.8 ± 14.6 years old, *p* < 0.001), had a lower proportion of females (69.6 vs. 78.3%, *p* = 0.029), longer RA duration (11.5 ± 15.2 vs. 7.3 ± 12.0 years, *p* = 0.003), lower body mass index (BMI) (20.9 ± 4.2 vs. 22. ± 3.9, *p* = 0.002), higher incidence of ILD (44.4 vs. 12.4%, *p* < 0.001), higher serum level KL-6 (485 ± 373 vs. 387 ± 442 U/ml, *p* < 0.001), and lower eGFR (84.2 ± 28.3 vs. 90.4 ± 28.9 ml/min/1.73m^2^, *p* = 0.014). However, no significant difference was found in DAS28-CRP, SDAI, use of MTX, glucocorticoids, other csDMARDs, and bDMARDs/tsDMARDs between AD + and AD– groups.

Regarding the four subgroups (Fig. 2, Table 1), ILD–AD– was significantly younger than the other three subgroups. There were fewer females in ILD + AD + than ILD–AD–. RA duration was longer in ILD + AD + than ILD + AD–, while BMI was higher in ILD + AD– and ILD–AD– than ILD–AD + . Diabetes was more frequent in ILD + AD + than ILD–AD–. No significant difference was found in DAS28-CRP or SDAI scores among the four subgroups. Use of MTX was less frequent in ILD + AD + and ILD + AD– than ILD–AD + and ILD–AD–. No significant difference was found in use of glucocorticoids, bDMARDS/tsDMARDs, and csDMARDs among the subgroups. There was no significant difference in type of ILD between ILD + AD + and ILD + AD– subgroup (UIP; 34.2 vs. 18.4%, *p* = 0.067) or AD between ILD + AD + and ILD–AD + subgroup (bronchiolitis; 38.2 vs. 52.6%, *p* = 0.066).

### Patient characteristics at final observation

**Fig. 3:**
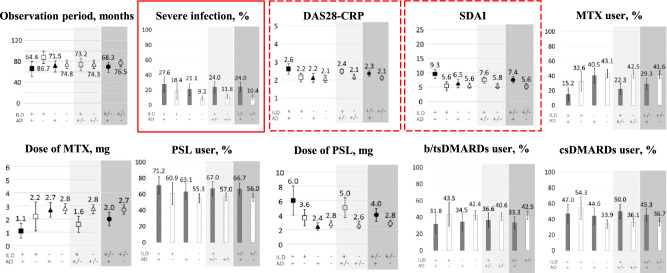
Patient characteristics at final observation. Characteristics of patient groups at final observation including proportions, means, and 95% confidence intervals. Abbreviations: PSL (prednisolone), MTX (methotrexate), bDMARDs/tsDMARDs (biologic disease modified anti-rheumatic-drugs / targeted synthetic DMARDs), csDMARDs (conventional synthetic DMARDs), DAS28-CRP (disease activity score-28 using C-reactive protein), SDAI (simplified disease activity index).

Clinical characteristics of patients at final observation are summarized in Fig. 3 and Table 2. The observation period did not significantly differ between ILD + and ILD– groups or AD + and AD– groups. In all four groups, DAS28-CRP and SDAI scores at final observation had significantly decreased compared to baseline (*p* < 0.05).

**Table 2:** Comparison of clinical characteristics at final observation.

	Total	Binary groups		*ILD* + *vs. ILD*–	Binary groups		*AD* + *vs. AD*–	Subgroups				*Inter-subgroup*
	All	ILD +	ILD–		AD +	AD–		ILD + AD +	ILD + AD–	ILD–AD +	ILD–AD–	
	n = 569	n = 125	n = 444	*p*	n = 171	n = 398	*p*	n = 76	n = 49	n = 95	n = 349	p*
Observational periods, month, mean (SD)	73.9 (57.3)	73.2 (58.7)	74.3 (57.1)	0.813	68.3 (56.1)	76.5 (57.8)	0.134	64.4 (59.0)	86.7 (56.2)	71.5 (53.8)	75.0 (58.0)	0.146
Severe infection, n (%)	82 (14.4)	30 (24.0)	52 (11.8)	< 0.001	41 (24.0)	41 (10.4)	< 0.001	21 (27.6)	9 (18.4)	20 (21.1)	32 (9.2)	< 0.001^c, f^
Respiratory infection, n (%)	53 (9.3)	22 (17.6)	31 (7.0)	< 0.001	30 (17.5)	23 (5.8)	< 0.001	16 (21.1)	6 (12.2)	14 (14.7)	17 (4.9)	0.015 ^c, f^
Disease activity of RA												
DAS28-CRP, mean (SD)	2.1 (1.0)	2.4 (1.0)	2.1 (1.0)	0.015	2.3 (1.1)	2.1 (1.0)	0.024	2.6 (1.1)	2.2 (0.9)	2.2 (1.0)	2.0 (1.0)	0.028^c^
SDAI, mean (SD)	6.1 (7.1)	7.6 (6.4)	5.8 (7.2)	0.034	7.4 (7.0)	5.6 (7.1)	0.036	9.3 (7.1)	5.6 (5.0)	6.5 (6.9)	5.6 (7.3)	0.044^c^
SJC, mean (SD)	1.2 (2.7)	1.7 (3.0)	1.1 (2.6)	0.013	1.6 (3.0)	1.0 (2.6)	0.009	2.2 (3.7)	1.1 (1.6)	1.3 (2.3)	1.0 (2.7)	0.020^c^
TJC, mean (SD)	1.1 (2.4)	1.1 (1.6)	1.1 (2.6)	0.398	1.1 (1.8)	1.1 (2.6)	0.423	1.4 (1.9)	0.7 (1.2)	0.9 (1.8)	1.2 (2.8)	0.179
EGA, mm, mean (SD)	23 (19.8)	25.1 (19.3)	22.5 (20.0)	0.274	24.2 (19.3)	22.6 (20.1)	0.385	25.9 (18.4)	24.3 (20.6)	23.3 (19.9)	22.3 (20.1)	0.643
PGA, mm, mean (SD)	14.9 (13.9)	15.5 (12.8)	14.8 (14.1)	0.53	16.1 (12.5)	14.5 (14.3)	0.106	17.9 (12.3)	12.8 (13.0)	15.2 (12.7)	14.7 (14.5)	0.237
CRP, mg/dl, mean (SD)	1.0 (2.4)	1.4 (2.8)	0.9 (2.2)	< 0.001	1.4 (3.1)	0.8 (2.0)	< 0.001	1.8 (3.4)	0.8 (1.4)	1.1 (2.8)	0.8 (2.1)	< 0.001^c^
Death, n (%)	26 (4.6)	8 (6.4)	18 (4.1)	0.33	10 (5.8)	16 (4.0)	0.383	6 (7.9)	2 (4.1)	4 (4.2)	14 (4.0)	0.858
Treatments												
MTX user, n (%)	188 (38.1)	25 (22.3)	161 (42.5)	< 0.001	44 (29.3)	142 (41.6)	0.01	10 (15.2)	15 (32.6)	34 (40.5)	127 (43.1)	< 0.001^b, c^
Dose of MTX, mg, mean (SD)	2.5 (3.6)	1.6 (3.3)	2.8 (3.6)	< 0.001	2.0 (3.3)	2.7 (3.7)	0.023	1.1 (2.7)	2.2 (4.0)	2.7 (3.6)	5.1(6.7)	0.001^b, c^
PSL user, n (%)	292 (59.2)	75 (67.0)	216 (57.0)	0.059	100 (66.7)	191 (56.0)	0.027	47 (71.2)	28 (60.9)	53 (63.1)	163 (55.3)	0.091
Dose of PSL, mg, mean (SD)	3.2 (5.3)	5.0 (7.4)	2.6 (4.3)	< 0.001	4.0 (6.4)	2.8 (4.7)	0.009	6.0 (9.0)	3.6 (3.7)	2.4 (2.1)	2.8 (3.6)	< 0.001^b, c^
Cumulative glucocorticoid dose, mg, mean (SD)	5731 (7627)	6963 (8769)	5405 (7261)	0.068	5674 (7723)	5777 (7607)	0.770	6481 (9274)	7727 (7937)	5029 (6188)	5484 (7518)	0.223
bDMARDs/tsDMARDs user, n (%)	197 (40)	41 (36.6)	154 (40.6)	0.444	50 (33.3)	145 (42.5)	0.055	21 (31.8)	20 (43.5)	29 (34.5)	125 (42.4)	0.255
TNFi, n (%)	75 (15.2)	9 (8.0)	64 (16.9)	0.023	14 (9.3)	59 (17.4)	0.022	4 (6.1)	5 (10.9)	10 (11.9)	54 (18.4)	0.052
IL-6i, n (%)	58 (11.8)	12 (10.7)	46 (12.1)	0.682	14 (9.3)	44 (12.9)	0.259	5 (7.6)	7 (15.2)	9 (10.7)	37 (12.5)	0.6
CTLA-4Ig, n (%)	38 (7.7)	12 (10.7)	26 (6.9)	0.18	14 (9.3)	24 (7.0)	0.381	7 (10.6)	5 (10.9)	7 (8.3)	19 (6.4)	0.535
tsDMARDs, n (%)	26 (5.3)	8 (7.1)	18 (4.7)	0.338	8 (5.3)	18 (5.3)	1	5 (7.6)	3 (6.5)	3 (3.6)	15 (5.1)	0.715
csDMARDs user, n (%)	194 (39.4)	56 (50.0)	137 (36.1)	0.008	68 (45.3)	125 (36.7)	0.07	31 (47.0)	25 (54.3)	37 (44.0)	100 (33.9)	0.052
BUC, n (%)	32 (6.5)	8 (7.1)	24 (6.3)	0.827	12 (8.0)	20 (5.9)	0.377	4 (6.1)	4 (8.7)	8 (9.5)	16 (5.4)	0.522
IGU, n (%)	30 (6.1)	3 (2.7)	27 (7.1)	0.114	9 (6.0)	21 (6.2)	1	3 (4.5)	0 (0)	6 (7.1)	21 (7.1)	0.269
SASP, n (%)	25 (5.1)	11 (9.8)	14 (3.7)	0.01	11 (7.3)	14 (4.1)	0.134	6 (9.1)	5 (10.9)	5 (6.0)	9 (3.1)	0.146
Tac, n (%)	135 (27.4)	39 (34.8)	95 (25.1)	0.042	44 (29.3)	90 (26.4)	0.5	18 (27.3)	21 (45.7)	26 (31.0)	69 (23.4)	0.015^e^
CAM, n (%)	40 (8.1)	13 (11.6)	27 (7.1)	0.13	22 (14.7)	18 (5.3)	< 0.001	11 (16.7)	2 (4.3)	11 (13.1)	16 (5.4)	0.005^c^
ST, n (%)	88 (17.9)	28 (25.0)	59 (15.6)	0.022	28 (18.7)	59 (17.4)	0.726	17 (25.8)	11 (23.9)	11 (13.1)	48 (16.3)	0.134

p*, p value from overall statistical comparison between four subgroups before post hoc pairwise comparisons. P-values from post hoc pairwise comparisons with Bonferroni’s correction are provided in the manuscript. a; ILD + AD + group vs ILD + AD- group, b; ILD + AD + group vs ILD-AD + group, c; ILD + AD + group vs ILD-AD- group, d; ILD + AD- group vs ILD-AD + group, e; ILD + AD- group vs ILD-AD- group, f; ILD-AD + group vs ILD-AD- group.

Abbreviations: ILD, interstitial lung disease; AD, airway disease; SD, standard deviation; RA, rheumatoid arthritis; BMI, body mass index; RF, rheumatoid factor; anti-CCP Abs, anti-cyclic citrullinated peptide antibodies; eGFR, estimated glomerular filtration rate; DAS28, disease activity score for 28 joints; CRP, C-reactive protein; SDAI, simplified disease activity index; SJC, swollen joint count; TJC, tender joint count; EGA, evaluator global assessment; PGA, patient global assessment; MMP-3, matrix metalloproteinase-3; MTX, methotrexate; PSL, prednisolone; DMARDs, disease modified anti-rheumatic-drugs; bDMARDs, biologic DMARDs; tsDMARDs, targeted synthetic DMARDs; csDMARDs, conventional synthetic DMARDs; TNFi, TNF inhibitor; IL-6i, IL-6 receptor inhibitor; CTLA-4Ig, cytotoxic T lymphocyte antigen-4 Immunoglobulin; BUC, bucillamine; IGU, iguratimod; SASP, salazosulfapyridine; Tac, Tacrolimus; CAM, Clarithromycin; ST, sulfamethoxazole/trimethoprim.

ILD + group, compared to ILD– group, had higher DAS28-CRP (2.4 ± 1.0 vs. 2.1 ± 1.0, *p* = 0.014) and SDAI scores (7.6 ± 6.4 vs. 5.8 ± 7.2, *p* = 0.034). Use of MTX was less common (22.3 vs. 42.5%, *p* < 0.001; 1.6 ± 3.3 vs. 2.8 ± 3.6 mg, *p* < 0.001) while glucocorticoid usage was higher (67.0 vs. 57.0%, *p* = 0.057; 5.0 ± 7.4 vs. 2.6 ± 4.3 mg, *p* < 0.001). Glucocorticoid dose at final observation did not significantly differ from that at baseline in ILD + group (*p* = 0.975), whereas it decreased in ILD– group (*p* < 0.001). The decrease in glucocorticoid dose was less pronounced in ILD + group than ILD– group (*p* = 0.006). Use of bDMARDs/tsDMARDs was similar between both groups.

AD + group, compared to AD– group, had higher DAS28-CRP (2.3 ± 1.1 vs. 2.1 ± 1.0,* p* = 0.024) and SDAI scores (7.4 ± 7.0 vs. 5.6 ± 7.1,* p* = 0.036). Use of MTX was less common (29.3 vs. 41.6%, *p* = 0.011; 2.0 ± 3.3 vs. 2.7 ± 3.7 mg, *p* = 0.023) while glucocorticoid usage was higher (66.7 vs. 56.0%, *p* = 0.026; 4.0 ± 6.4 vs. 2.8 ± 4.7 mg, *p* = 0.009). Glucocorticoid dose at final observation was not decreased from that at baseline in the AD + group (*p* = 0.129), while it decreased in the AD– group (*p* < 0.001). In contrast to ILD + and ILD– groups, there was no difference in decrease of glucocorticoid dose between AD + and AD– groups (*p* = 0.193). Use of bDMARDs/tsDMARDs tended to be lower in AD + group than AD − group, albeit not significantly. Use of csDMARDs were similar between both groups.

Among the four subgroups (Fig. 3, Table 2), there was no difference in observation period. ILD + AD + subgroup, compared to ILD–AD– subgroup, had higher DAS28-CRP (2.6 ± 1.1 vs. 2.0 ± 1.0,* p* = 0.028) and SDAI scores (9.3 ± 7.1 vs. 5.6 ± 7.3, *p* = 0.031). Use of MTX was less common (15.2 vs. 43.1%, *p* < 0.001; 1.1 ± 2.7 vs. 5.1 ± 6.7 mg, *p* = 0.001) while glucocorticoid usage was higher (71.2 vs. 55.3%, *p* = 0.101; 6.0 ± 9.0 vs. 2.8 ± 3.6 mg, *p* < 0.001). Glucocorticoid dose at final observation was not decreased from that at baseline in ILD + AD + and ILD + AD– subgroups (*p* = 0.966 and 0.909), whereas it was in ILD–AD + and ILD–AD– subgroups (*p* = 0.024 and < 0.001). Use of bDMARDs/tsDMARDs was similar among the four subgroups.

### Association of ILD and AD with final RA activity

As the interaction between ILD and AD did not significantly impact final RA activity in the pilot ANCOVA, it was excluded from the final analysis. When adjusting for age, sex, Ln(DAS28-CRP at baseline), and other factors known to affect RA disease activity, the final analysis revealed significant associations between age and ILD with Ln(DAS28-CRP at final observation), whereas AD showed no significant association (Table 3a). The estimated marginal (EM) mean difference in Ln(DAS28-CRP at final observation) between ILD + and ILD– groups was 0.279 (95% CI 0.074–0.484), corresponding to an EM geometric mean ratio of 1.322 (1.077–1.623) in the original linear scale. Similarly, age and ILD were significantly associated with Ln(SDAI at final observation), but AD was not (Table 3b). The EM mean difference in Ln(SDAI at final observation) between ILD + and ILD– groups was 0.757 (95% CI 0.079–1.434), equivalent to an EM geometric mean ratio of 2.132 (1.082–4.195) in the linear scale.

**Table 3:** Factorial analysis of covariance for disease activity score for 28 joints and simplified disease activity index.

a. DAS28-CRP	Analysis 1		Analysis 2	
	β	95%CI	β	95%CI
Age	0.007	0.002–0.012	0.007	0.001–0.012
Female	− 0.058	− 0.237–0.120	− 0.064	− 0.242–0.114
ILD	0.194	− 0.079–0.468	0.279	0.074–0.484
AD	− 0.07	− 0.267–0.126	− 0.023	− 0.192–0.145
ILD*AD	0.186	− 0.210–0.581	not included	
Ln (DAS28− CRP at baseline)	0.06	− 0.075–0.196	0.066	− 0.069–0.201
RF and/or anti− CCP Abs	− 0.028	− 0.206–0.150	− 0.043	− 0.218–0.132
MTX dose	0.009	− 0.010–0.028	0.009	− 0.010–0.028
PSL dose	0.008	− 0.013–0.029	0.008	− 0.013–0.029
bDMARDs and/or tsDMARDs	− 0.07	− 0.214–0.073	− 0.069	− 0.213–0.074

	ILD +	ILD–	Difference/ratio	ILD +	ILD–	difference/ratio
Ln (DAS28-CRP at final observation						
Estimated Marginal means	0.890	0.603	0.287† (0.082–0.493)	0.903	0.624	0.279† (0.074–0.484)
DAS28-CRP at final observation						
Estimated Marginal geometric means	2.435	1.828	1.332‡ (1.085–1.637)	2.467	1.866	1.322‡ (1.077–1.623)

b. SDAI	Analysis 1		Analysis 2	
	β	95%CI	β	95%CI
Age	0.017	0.001–0.033	0.016	0.000–0.032
Female	− 0.121	− 0.667–0.425	− 0.132	− 0.675–0.411
ILD	0.559	− 0.355–1.473	0.757	0.079–1.434
AD	− 0.287	− 0.891–0.316	− 0.197	− 0.730–0.336
ILD*AD	0.437	− 0.912–1.787	not included	
Ln (SDAI at baseline)	− 0.023	− 0.213–0.166	− 0.016	− 0.203–0.171
RF and/or anti-CCP Abs	− 0.076	− 0.616–0.464	− 0.108	− 0.637–0.422
MTX dose	0.034	− 0.023–0.092	0.032	− 0.024–0.089
PSL dose	− 0.024	− 0.070–0.022	− 0.025	− 0.071–0.020
bDMARDs and/or tsDMARDs	− 0.196	− 0.664–0.272	− 0.201	− 0.666–0.265

	ILD +	ILD–	difference/ratio	ILD +	ILD–	difference/ratio
Ln(SDAI at final observation						
Estimated Marginal means	2.097	1.320	0.777† (0.095–1.460)	2.115	1.358	0.757† (0.079–1.434)
SDAI at final observation						
Estimated Marginal geometric means	8.142	3.743	2.175‡ (1.100–4.306)	8.29	3.888	2.132‡ (1.082–4.195)

### Impact of ILD and AD on infectious events

**Fig. 4:**
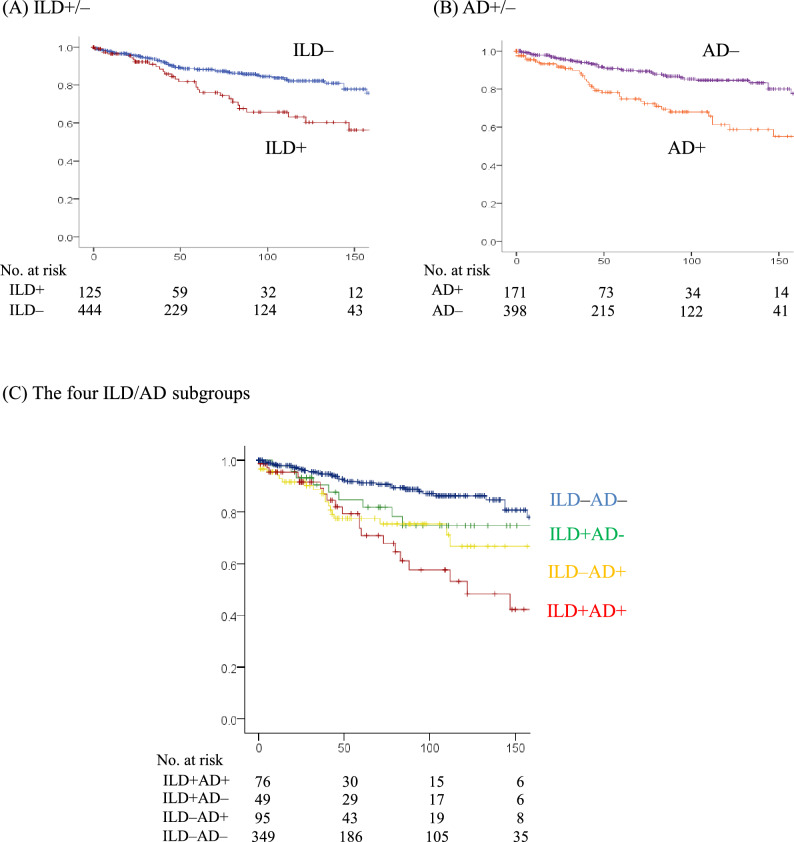
Cumulative rate of total infectious events. Kaplan–Meier analysis with log-rank test showing significant differences in total infectious events-free rate (EFR) between (**A**) interstitial lung disease (ILD) + and ILD– group (*p* < 0.001), and between (**B**) airway disease (AD) + and AD– group (*p* < 0.001). (**C**) Survival curves for the four ILD/AD groups.

Severe infections events were observed in 82 cases: pneumonia, 53 cases; herpes zoster, 8 ; urinary tract infection, 6. Severe infectious events were more frequent in ILD + group than ILD– group (24.0% vs. 11.8%, *p* < 0.001) and in AD + group than AD– group (24.0% vs. 10.4%, *p* < 0.001) (Fig. 3, Table 2). As in Fig. 4A, the Kaplan–Meier curve of infectious events-free rate (EFR) for ILD + and ILD– groups initially displayed no significant difference. However, over time, the former exhibited a gradual decrease, with the between-group difference over the entire follow-up period statistically significant (log-rank test, *p* < 0.001). In contrast, the Kaplan–Meier curve of infectious EFR in AD + group was lower than that in AD– group from the beginning of the observation period (log-rank test,* p* < 0.001) (Fig. 4B). Similarly, respiratory infection events were more frequent in ILD + group (17.6% vs. 7.0%, *p* < 0.001) and AD + group (17.5% vs. 5.8%, *p* < 0.001), and the Kaplan–Meier curve of respiratory infectious EFR in ILD + group (log-rank test, *p* < 0.001) and AD + group was lower (log-rank test, *p* < 0.001).

Among the four subgroups, severe infectious events were more frequent in ILD + AD + and ILD–AD + subgroups than ILD–AD– subgroup (27.6 vs. 9.2%, *p* < 0.001; 21.1 vs. 9.2%, *p* = 0.016) (Fig. 3, Table 2). Kaplan–Meier analysis and log-rank test revealed infectious EFR in ILD + AD + subgroup was lower than that in ILD + AD– subgroup, and that in ILD–AD + subgroup was lower than that in ILD–AD– subgroup (*p* < 0.001) (Fig. 4C). No significant difference was found in infectious EFR between ILD + AD + and ILD–AD + subgroups, and between ILD + AD– and ILD–AD– subgroups (*p* = 0.057) (Fig. 4C).

The first Cox regression analysis was performed with ILD, AD, and ILD*AD (their interaction), adjusted for age, sex, and BMI. AD, age and BMI significantly affected time to infectious events, while ILD and ILD*AD did not (Table 4). Considering the potential time-dependent effect of ILD, we conducted a Cox regression analysis including a time-dependent covariate. The interaction between ILD and follow-up duration ≥ 50 months showed a significant association with increased risk of infectious events (Hazard ratio 3.249, 95% CI 1.194–2.840), while ILD alone was not significant (Hazard ratio 1.139, 95% CI 0.602–2.157). AD remained significant associated with infection risk (HR 1.744, 95%CI 1.054–2.885), as did age (HR 1.037, 95%CI 1.016–1.059) and BMI (HR 0.927, 95%CI 0.871–0.988) (Table 4). To assess whether the interaction between ILD and follow-up period of ≥ 50 months, and AD were independent of cumulative glucocorticoid dose, we divided patients into two groups based on cumulative dose and included in Cox regression analysis alongside a reference group of patients who did not receive glucocorticoids. Sensitivity analysis was performed using split points at every 1,000 mg increment to determine the optimal threshold. A significant effect on severe infection incidence was observed at cumulative doses ≥ 16,000 mg (HR 2.681, 95%CI 1.023–7.021). No significant changes in HR or 95%CI were observed for ILD*[time =  > 50] or AD.in HR or 95% C.I. for ILD*[time =  > 50], or for AD.

**Table 4:** Multivariate Cox regression analysis with/without time dependent covariates for the total infectious events.

	Analysis 1		Analysis 2		Analysis 3	
	HR	95%CI	HR	95%CI	HR	95%CI
Female	0.946	0.562–1.592	0.947	0.562–1.593	0.965	0.573–1.627
Age	1.036	1.014–1.058	1.037	1.016–1.059	1.046	1.022–1.069
ILD	2.042	0.948–4.401	1.139	0.602–2.157	1.111	0.587–2.101
AD	1.970	1.065–3.646	1.744	1.054–2.885	1.731	1.046–2.865
BMI	0.927	0.871–0.988	0.927	0.871–0.988	0.926	0.869–0.987
ILD x AD	0.758	0.281–2.050	not included		Not included	
ILD x [Time > = 50]	Not included		3.249	1.194–8.840	3.520	1.288–9.619
Cum. GC = 0 mg	Not included		Not included		Reference	
Cum. GC > 0- 16,000 mg	Not included		Not included		1.284	0.545–3.027
Cum. GC > 16,000 mg	Not included		Not included		2.681	1.023–7.021

Analysis 1 included Age, Female, ILD, AD, and ILD*AD as fixed variates, as well as BMI, Diabetes Mellitus (y/n), MTX (y/n), PSL (y/n), and b/ts DMARDs (y/n) as stepwise selection variates. Analysis 2 included the same as Analysis 1, except ILD*AD, and ILD*[tune >—50 month] was added. Analysis 3 included the same as Analysis 2, and cumulative glucocorticoid dose was added. [Time >  = 50], Time-dependent covariate indicating whether the time since baseline has reached 50 months; Cum. GC, cumulative glucocorticoid dose.

## Discussion

In previous research on RA-associated ILD, studies have included cases with coexisting AD, and vice versa. Consequently, the individual and interactive effects of ILD and AD on prognosis, disease activity, and infection risk in RA patients, have remained unclear. In this study, we investigated the isolated and combined effects of ILD and AD on the long-term outcomes of RA disease activity and infection risk within the same cohort over the same period.

Prior studies have indicated that pulmonary involvement can complicate RA treatment and exacerbate disease activity. A recent Japanese study revealed around 10% of difficult-to-treat RA cases were linked to comorbidities, with lung conditions the most prevalent^27^. RA patients with lung involvement may face limitations in using MTX, the main drug in RA treatment, due to risks of MTX-related pneumonitis and ILD deterioration^28^. Consequently, the therapeutic effectiveness of bDMARDs in RA patients with lung involvement may be compromised, leading to RA management challenges^27^. In our study, ILD + group had fewer MTX users than ILD– group at baseline. At final observation, ILD + group continued to have fewer MTX users, but there was an increasing trend in the use of prednisolone (PSL). Conversely, AD + group did not exhibit a significant difference in DMARDs usage compared to AD– group at baseline. At final observation AD + group had fewer MTX users and a higher use of PSL. Patients with RA and ILD had received less MTX and more PSL. RA patients with AD were treated like those without AD. In treatment provided by our department, patients with RA and ILD or AD consistently received less MTX, with treatment primarily centered around PSL. This choice of treatment strategy may be attributed to the lack of widely-accepted consensus or recommendations for treatment of patients with both RA and AD^29,30^.

In this study, we conducted covariate analysis adjusted for baseline MTX and PSL doses, the use of bDMARDs/tsDMARDs and Ln(DAS28-CRP at baseline). We found ILD was independently associated with inadequate control of RA disease activity, and presence of AD did not significantly impact disease activity implying the influence of ILD on disease activity was not mediated by the type or amount of DMARDs, at least at baseline.

We revealed that AD independently increased the risk of serious infections, whereas ILD increased the risk of infection after 50 months. While ILD and AD have been reported as risk factors for infections^2,18^, our study is the first to show they independently increased infection risk. HR for serious infection after 50 months was high for ILD at 3.701 and low for AD at 1.744. Explaining this magnitude difference and their timing is challenging, as it may not be attributed to a single cause. The significantly higher dose of PSL prescribed in the ILD + group at the final observation may have affected the differences in effects and timing of serious infection^31^, contrary to the recommendation that glucocorticoid use should be reduced and stopped as early as possible to avoid steroid toxicity, including infection risk^28,32^. While short-term glucocorticoid use is considered safe and effective in RA treatment, long-term use poses an infection risk^31^. Early as possible reduction or discontinuation is recommended^28,32^. In ILD + group, since RA control tended to become insufficient, achieving adequate glucocorticoid reduction might have been impossible. Some cases of RA with ILD might have experienced worsening of ILD during the long-term clinical course, leading to serious infections. One study reported some rheumatologists have insufficient awareness of AD as an infection risk factor^21^. The treatment in AD + group may have overlooked infection risk. In the long-term clinical course of RA, since ILD and AD are independent infection risk factors, establishment of a safer treatment strategy is required.

ILD + AD + is relatively common in RA, with poorer RA disease activity control and a greater susceptibility to infection than the other groups. In our study, ILD + AD + group accounted for 13.4% of the study population. The prevalence was similar to that of ILD + AD– and ILD–AD + groups. Approximately half of the patients with ILD had AD. ILD + AD + group exhibited higher final RA disease activity than the other groups. In ILD + AD + group, it was suspected that MTX use was less common, leading to inadequate disease activity control, insufficient steroid reduction, or missed opportunities for steroid discontinuation. A previous study analyzing respiratory infections in RA patients treated with bDMARDs over a mean observational period of 7.4 years^20^ found cumulative incidences of respiratory infections in ILD + AD + (n = 6), ILD + AD– (n = 29) and ILD–AD + groups (n = 76) were 23.6, 11.3, and 17.6 per 1000 patient-years, respectively. Although the number of patients in ILD + AD + group was small, this report suggested ILD + AD + patients require equal or more attention to infection risk than those in ILD + AD– and ILD–AD + groups. In the present study, although no interaction between ILD and AD was observed regarding RA disease activity and infection, ILD + AD + group was found to be a specific group in which these independent impacts of ILD and AD overlapped. Therefore, special attention is needed in treatment strategy and prevention of infection in ILD + AD + group.

Our study has some limitations. It was a retrospective study from a single-center, and included only 79% of the RA population. Due to the limited number of cases in our retrospective hospital cohort, the subgroup sample sizes in this study may have been underpowered to detect the modest effect size attributable to the [ILD*AD] interaction. Some patient cases had missing data, including RF and anti-CCP antibody measurements. Lung involvement was diagnosed based on CT findings, without considering respiratory symptoms, pulmonary function, or pathological findings.

In summary, our study revealed that ILD alone is independently associated with future RA disease activity, indicating inadequate disease control in RA patients with ILD compared to those without ILD. Also, AD is associated with infections leading to a higher frequency of hospitalization of RA patients with AD. After 50 months, ILD becomes an independent infection risk factor. There is no interaction between ILD and AD, but patients with both lung involvements are at increased risk of infections. Therefore, for RA patients, it is crucial to individually assess the presence of these two lung disorders, evaluate infection risks, and establish safe alternative treatments to effectively control RA while avoiding infections.

## Supplementary Information

Supplementary Information.

## Data Availability

Raw data were generated at Kanazawa University Hospital. Derived data supporting the findings of this study are available from the corresponding author M.Y. on request.
