# Supplementary material for: Differential impacts of interstitial lung disease and airway disease on rheumatoid arthritis disease activity and infection
Source: Sci Rep. 2025 Nov 13;15:39757. doi: 10.1038/s41598-025-23080-1 (PMC12615712; doi:10.1038/s41598-025-23080-1)
Supplement: Supplementary file 1 — Supplementary Information. [file 41598_2025_23080_MOESM1_ESM.docx]

**Supplemental Methods**

**Statistical analyses**

Continuous variables were presented as (arithmetic) mean ± standard deviation, except when specified otherwise. Significant differences in continuous variables between two independent groups were evaluated using Mann-Whitney U-test. For comparisons among four groups, the Kruskal-Wallis test was applied, followed by pairwise Mann-Whitney U test with Bonferroni’s correction.

Categorical variables were presented as counts and percentages, and comparisons of proportions were performed using Pearson's chi-square test or Fisher’s exact test, as appropriate Post hoc pairwise z-tests with Bonferroni correction were used for comparisons among four groups.

Wilcoxon signed-rank sum test was used to compare glucocorticoid doses at baseline and at final observation.

Factorial analysis of covariance (ANCOVA) was used to determine the impacts of ILD, AD, and their interaction on RA disease activity at final observation. Multivariate adjustments were performed for known factors associated with RA disease activity, including age, sex, positivity of either or both rheumatoid factor and anti-cyclic citrullinated peptide (CCP) antibodies, RA disease activity at baseline, methotrexate dose, glucocorticoid dose, and bDMARDs/tsDMARDs use [33, 34].

Absence of multicollinearity was determined as Spearman’s rank correlation coefficient between any combination of independent variables <0.7. Normality of the residual distribution was assessed graphically by histogram and Q-Q plots for all patients and each group, with the results of Shapiro-Wilk test, skewness, and kurtosis as additional information. When normality was in question, logarithmic transformation of the dependent variable was considered. Linear regression between dependent variables and each covariate was assessed graphically by scatter diagram for all patients and each group, and similarity in slopes of these regressions was confirmed. The homoscedasticity of residuals was also verified for all patients and for each group by visual inspection and by Levene's test. The presence of outlier was checked with histograms of studentized residuals, centralized leverage values, and Cook’s distance values.

Estimated marginal means in dependent variable for ILD+ and ILD– groups, as well as their difference, and for AD+ and AD– and their difference were described with 95% confidence intervals. Estimated marginal (arithmetic) means of log-transformed variable were back-transformed to geometric means in original scale, and their differences to geometric mean ratios.

The Kaplan-Meier method was used to estimate the cumulative infectious event-free rate, and stratified log-rank test to assess the difference between groups. Cox’s proportional hazard model was applied to determine the impact of baseline ILD, AD, and their interaction, on the time to infectious events requiring hospitalization. We added several baseline factors which had been already known to be the risks of infections, e.g., age, sex, body mass index (BMI), diabetes, MTX use, glucocorticoid use, and bDMARDs/tsDMARDs use, cumulative glucocorticoid doses [35–39], for multivariate adjustment. The hazard proportionality was assessed by visual inspection of the Kaplan-Meier curve and log(-log(Survival)) plot. When the proportionality was in question, Cox’s analysis with time dependent covariates was considered when appropriate.

The collected data was analyzed using SPSS (Version 27.0, IBM Corp. Armonk, NY, USA) with p <0.05 deemed significant. Hazard ratios (HR) were expressed with 95% confidence intervals (CI).

**References for Supplemental Methods**

33 Shpatz R, Braun-Moscovici Y, Balbir-Gurman A. ACPA Antibodies Titer at the Time of Rheumatoid Arthritis Diagnosis Is Not Associated with Disease Severity. Isr Med Assoc. 23, 646–50 (2021).

34 Shadick NA, Gerlanc NM, Frits ML et al. The longitudinal effect of biologic use on patient outcomes (disease activity, function, and disease severity) within a rheumatoid arthritis registry. Clin Rheumatol. 38, 3081–92 (2019).

35 Doran MF, Crowson CS, Pond GR, O'Fallon WM, Gabriel SE. Predictors of infection in rheumatoid arthritis. Arthritis Rheum. 46, 2294–300 (2002).

36 Thomas K, Lazarini A, Kaltsonoudis E et al. Incidence, risk factors and validation of the RABBIT score for serious infections in a cohort of 1557 patients with rheumatoid arthritis. Rheumatology. 60, 2223–30 (2021).

37 Mori S, Yoshitama T, Hidaka T et al. Comparative risk of hospitalized infection between biological agents in rheumatoid arthritis patients: A multicenter retrospective cohort study in Japan. PLoS One. e0179179 (2012).

38 Komano Y, Tanaka M, Nanki T et al. Incidence and risk factors for serious infection in patients with rheumatoid arthritis treated with tumor necrosis factor inhibitors: a report from the Registry of Japanese Rheumatoid Arthritis Patients for Longterm Safety. J Rheumatol. 38, 1258–64 (2011).

39 Leombruno JP, Einarson TR, Keystone EC. The safety of anti-tumour necrosis factor treatments in rheumatoid arthritis: meta and exposure-adjusted pooled analyses of serious adverse events. Ann Rheum Dis. 68, 1136–45 (2009).
